# *Streptococcus oralis* MitraClip endocarditis following a dental procedure: a case report

**DOI:** 10.1186/s12879-021-06565-y

**Published:** 2021-08-28

**Authors:** Aikaterini Papamanoli, Tahmid Rahman, Andreas P. Kalogeropoulos, Zeena Lobo, Paul Diggs, Anne Hamik, George Psevdos

**Affiliations:** 1 Division of Infectious Diseases, Stony Brook University Hospital, Stony Brook, NY USA; 2 Division of Cardiology, Northport Veterans Affairs Medical Center, Northport, NY USA; 3 Division of Cardiology, Stony Brook University Hospital, Stony Brook, NY USA; 4 Division of Infectious Diseases, Northport Veterans Affairs Medical Center, NY Northport, USA

**Keywords:** MitraClip, Infective endocarditis, Antibiotic prophylaxis, Dental procedures, Case report

## Abstract

**Background:**

Transcatheter edge-to-edge mitral valve repair using the MitraClip device is increasingly used for high surgical risk patients with severe mitral regurgitation (MR). Previous guidelines for infective endocarditis prophylaxis prior to dental procedures focused on high-risk patients, but without explicit recommendation for MitraClip recipients. We believe this could be the first reported case to identify *Streptococcus oralis* as the causative organism.

**Case presentation:**

An 87-year-old male with severe MR treated with two MitraClip devices three months prior to index admission, presented with worsening malaise and intermittent chills on a background of multiple comorbid conditions. The patient had dental work a month prior to presentation, including a root canal procedure, without antibiotic prophylaxis. Vitals were significant for fever and hypotension. On physical examination, there was a holosystolic murmur at the apex radiating to the axilla, bilateral pitting edema in the lower extremities, and elevated jugular venous pulsation. A transthoracic echocardiogram showed severe MR with a possible echodensity on the mitral valve, prompting a transesophageal echocardiogram, which demonstrated a pedunculated, mobile mass on the posterior leaflet of the mitral valve. Five blood cultures grew gram positive cocci in pairs and chains, later identified as *Streptococcus oralis*, with minimum inhibitory concentration to penicillin 0.06 mg/L. Initial empiric antibiotics were switched to ceftriaxone 2 gr daily and subsequent blood cultures remained negative. However, the patient developed pulmonary edema and worsening hemodynamic instability requiring vasopressors. As surgical risk for re-operation was considered prohibitive, the decision was made to continue medical management and comfort-directed care. The patient died a week later.

**Conclusions:**

Despite low incidence, infective endocarditis should be included in the differential among MitraClip recipients. The explicit inclusion of this growing patient population in the group requiring prophylaxis prior to dental procedures in the 2020 ACC/AHA valvular heart disease guidelines is an important step forward.

**Supplementary Information:**

The online version contains supplementary material available at 10.1186/s12879-021-06565-y.

## Background

Transcatheter edge-to-edge mitral valve repair using the MitraClip (Abbott Vascular, Santa Clara, CA) is becoming a more widely used treatment strategy for high surgical risk patients with severe mitral regurgitation (MR). The device is a small metal clip, made of cobalt–chromium covered with polypropylene fabric to promote tissue in-growth, which is inserted percutaneously. MitraClip reduces MRl clipping together a portion of the mitral valve leaflets. Indications for MitraClip will expand, as guidelines now include patients with heart failure and functional secondary MR, which creates a large pool of potential candidates for MitraClip [1, 2].

The reported infective endocarditis (IE) incidence after MitraClip is very low. In the COAPT study, where patients with heart failure and secondary MR received a MitraClip device, the rate of IE requiring surgery at 12 months post procedure was 0 % [3]. In a large German study of 13,575 patients, only 0.1–0.3 % of MitraClip recipients developed IE but it was a major factor for in-hospital mortality when it occurred [4].

Despite the low incidence of IE after the MitraClip procedure, ΙΕ should be considered in the differential among MitraClip recipients in the appropriate context, as this permanent device is clipping the mitral valve leaflets. Although current IE prophylaxis guidelines prior to dental procedures focus on high-risk patients, there is no explicit recommendation for MitraClip recipients. Consequently, these cases may fall into a grey zone of expert opinion, creating a potential gap in the management of these patients.

We believe this is the first reported case to identify *Streptococcus oralis* as the causative organism for MitraClip IE, in a patient who had dental work without antimicrobial prophylaxis.

## Case presentation

An 87-year-old male with severe MR treated with transcatheter insertion of two MitraClip devices in November 2019, presented to the emergency department in February 2020 with 10 days of worsening malaise and intermittent chills, but no other associated symptoms. The patient had a background of nonischemic cardiomyopathy with preserved ejection fraction, paroxysmal atrial flutter, nonobstructive coronary artery disease, chronic kidney disease stage 3 (baseline creatinine of 1.6 mg/dL), chronic obstructive pulmonary disease, hypertension, and was recently under investigation for weight loss. The decision to treat the patient with a transcatheter procedure was based on severe heart failure symptoms refractory to initial medical therapy, with transthoracic and transesophageal echocardiography showing severe mitral regurgitation with torn chordae and posterior leaflet prolapse of the P2 segment, in a patient who was not deemed a good surgical candidate. Following the MitraClip procedure, the patient had improvement in cardiac symptoms and was in relatively good health. The post-procedural transesophageal echocardiogram showed only mild residual mitral regurgitation. Preprocedural dental consult showed no active dental infections. However, the patient had dental work a month prior to presentation, including a root canal procedure without antibiotic prophylaxis.

**Fig. 1:**
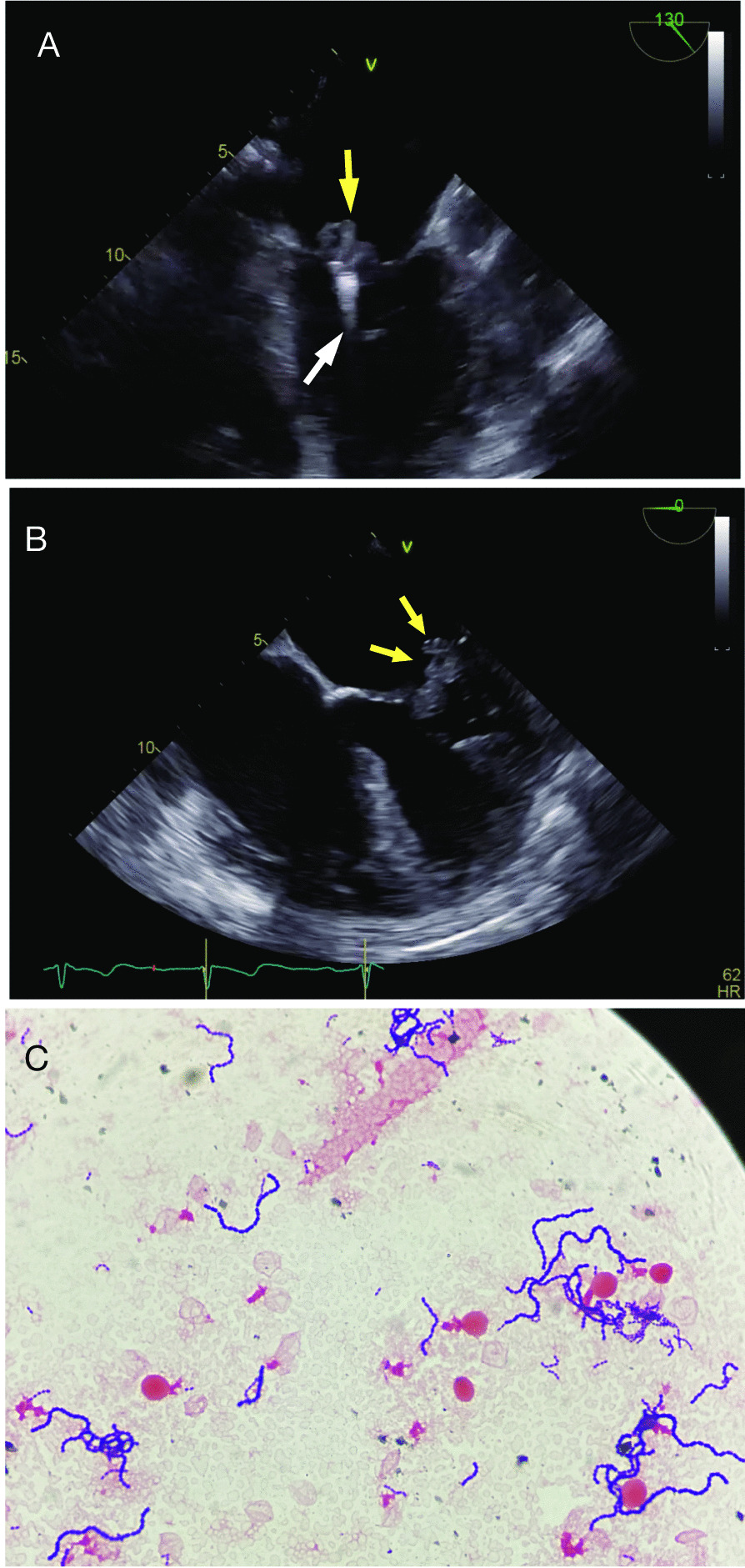
**A** Mid-esophageal long axis view on transesophageal echocardiogram (TEE) showing the MitraClip device (white arrow) and a large pedunculated mass consistent with vegetation (yellow arrow) on the atrial surface of the mitral valve. **B** Mid-esophageal 4-chamber TEE view showing a large vegetation (yellow arrows) on the atrial surface of the posterior mitral valve leaflet. **C** Gram positive cocci in chains seen on a gram stain of a positive blood culture

Upon arrival, the patient was alert and fully oriented, without signs of respiratory distress, and oxygen saturation 96 % on room air. However, he was febrile (38.6 °C) and hypotensive (blood pressure 81/45 mmHg). On physical examination, there was a loud holosystolic murmur at the apex radiating to the axilla, bilateral pitting edema in the lower extremities, and elevated jugular venous pulsation. Laboratory studies revealed elevated white blood cell count (20.5 K/µL with a left shift), creatinine (2.1 mg/dL), and lactic acid (2.4 mmol/L). An X-ray and computed tomography of the chest were unrevealing. A bedside transthoracic echocardiogram showed severe MR with a possible echodensity on the mitral valve. This finding prompted a transesophageal echocardiogram, which demonstrated a 2.0 × 0.8 cm pedunculated, mobile vegetation on the P2 segment of the posterior leaflet of the mitral valve on the MitraClip as evident from Fig. 1A, B and the transesophageal echocardiogram movie clips (Additional file 1: Video S1, Additional file 2: Video S2, Additional file 3: Video S3).

Five sets of blood cultures were collected during the first two days of admission and all grew gram positive cocci in pairs and chains which were later identified as *Streptococcus oralis* (Fig. 1C), by matrix-assisted laser desorption/ionization (MALDI) technique, with minimum inhibitory concentration (MIC) to penicillin 0.06 mg/L (Vitek^®^ 2, bioMerieux, Durham, NC). The patient was initially started on empiric antibiotics with vancomycin and cefepime, which were switched to ceftriaxone 2 gr once a day, when the blood culture results, and antibiotic sensitivities became available. Subsequent blood cultures remained negative. The patient did not develop signs or symptoms of a secondary lesion (e.g., stigmata of endocarditis) and no further systematic work up for metastatic lesions was performed. During the first 48 h of his admission, following fluid resuscitation, the patient developed pulmonary edema with worsening hemodynamic instability requiring vasopressor support. Although toxic shock syndrome was considered, as the patient had hypotension and renal impairment, there were no additional criteria for this syndrome, and thus his deterioration was felt to be secondary to cardiogenic rather that septic shock. Cardiothoracic surgery was consulted for potential removal of the clips and mitral valve replacement. However, because of multiple comorbidities, the patient was considered of prohibitive surgical risk. Therefore, the decision was made to continue medical management with intravenous ceftriaxone for 6 more weeks and possibly lifelong oral antibiotic suppression therapy. Despite a marginal improvement in clinical status with appropriate antibiotic therapy, the patient remained in a fragile hemodynamic and fluid management balance, struggling to control hypoperfusion, renal impairment, and elevated cardiac filling pressures. Eventually, palliative care discussions with the patient and the family led to the decision of comfort-directed care. The patient died a week later.

## Discussion and conclusions

Between 2003 (initial device approval in Europe) and 2020, approximately 20 cases of IE after MitraClip have been reported [5–13]. In a review of the first 12 cases, the pathogens were *Staphylococcus aureus* (N = 6), *Streptococci* (alpha-hemolytic N = 1, beta-hemolytic N = 1 with concomitant *S. aureus* infection), *Enterococcus faecalis* (N = 2), *Staphylococcus epidermidis* (N = 1), and unknown N = 2 [5]. A possible source of infection was reported in two cases with history of recurrent erysipelas and peripheral venous catheter infection, respectively, both caused by S. aureus. Most cases (N = 9) occurred within 12 months and 5 within 1 month of the procedure. Eight of the 12 patients were treated surgically despite high surgical risk, 3 of which relapsed [5]. In later reports where the microorganism was specified, *Bartonella henselae, Pseudomonas aeruginosa*, methicillin-resistant or unspecified *Staphylococcus aureus, Enterococcus faecalis, and Corynebacterium species* were isolated [6–13]. Of note, a dental condition was not mentioned in any previous report.

We believe this is the first reported case to identify *Streptococcus oralis* as the causative organism for MitraClip IE, with the caveat of a previous report of an alpha-hemolytic streptococcus that has not been further identified [5]. *S. oralis*, an alpha-hemolytic streptococcus, is a member of the normal human oral microbiota, capable of opportunistic pathogenicity. *S. oralis* has low virulence and is involved usually in subacute IE. Our patient had a root canal procedure 3 months after insertion of the two MitraClip devices without prophylactic antibiotics and presented one and a half month later with IE and septic shock. However, it is important to note that a definite causal relation between the invasive dental procedure and IE would be difficult to establish, as oral streptococcal IE can also result from daily dental activities.

The version of the European and American guidelines that was current at the time of the dental procedure of this patient limited the need for prophylactic antibiotics to high risk patients with prosthetic valve replacement or repair, those with congenital heart disease and shunts, previous IE, and cardiac transplant recipients [14–16]. The rationale was that (a) the estimated incidence of IE after dental procedures is very low and thus the number needed to treat with antibiotics to prevent 1 case would be very high; (b) oral bacteria are usually responsive to antibiotics; and (c) concerns about resistance and anaphylaxis with antibiotics. Although previously patients with transcatheter valve implants and those with surgical repairs using prosthetic materials like rings and chords were considered for prophylaxis in updated versions of the guidelines for valvular heart disease [17], transcatheter edge-to-edge mitral repairs were only explicitly discussed in the recently released 2020 ACC/AHA guideline for the management of patients with valvular heart disease [18]. Although prophylaxis for dental procedures in these patients received a IIa (moderate) recommendation in these guidelines, this seems to be appropriate considering the strength of the currently available evidence.

In conclusion, despite the low incidence of IE among MitraClip recipients, this diagnosis should always be included in the differential in the appropriate clinical setting. As oral bacteria including *S. oralis* can be involved in IE, we believe that the updated 2020 ACC/AHA valvular heart disease guidelines are a step towards the right direction and will alert caregivers for the appropriate management of this growing patient population prior to dental procedures. Ideally, these guidelines should be developed in collaboration with dental professional societies and appropriately disseminated to practicing dentists, as these frontline professionals are the ones that need to be aware of cardiac conditions that require appropriate antibiotic prophylaxis.

## Supplementary Information

**Additional file 1: Supplemental Video S1.** Transthoracic echocardiogram, apical 4-chamber view demonstrating severe central mitral regurgitation and a mass attached to the mitral valve leaflets near the MitraClip insertion point, protruding towards the left atrium.

**Additional file 2: Supplemental Video S2.** Transesophageal echocardiogram, mid-esophageal long-axis view demonstrating a mobile mass, consistent with vegetation, on the atrial surface of the mitral valve, near the MitraClip insertion point, at the P2 segment of mitral valve.

**Additional file 3: Supplemental Video S3.** Transesophageal echocardiogram, mid-esophageal 4-chamber view of the mass, which appears to be mobile and pedunculated.

## Data Availability

Not applicable.

## Abbreviations

IE: Infective endocarditis
MIC: Minimum inhibitory concentration
MR: Mitral regurgitation

## References

[1] Nishimura RA, Otto CM, Bonow RO, Carabello BA, Erwin JP, Guyton RA (2014). 2014 AHA/ACC guideline for the management of patients with valvular heart disease: a report of the American college of cardiology/American heart association task force on practice guidelines. J Am Coll Cardiol.

[2] Bonow RO, O’Gara PT, Adams DH, Badhwar V, Bavaria JE, Elmariah S (2020). 2020 Focused update of the 2017 ACC expert consensus decision pathway on the management of mitral regurgitation: a report of the American College of Cardiology Solution Set Oversight Committee. J Am Coll Cardiol.

[3] Stone GW, Lindenfeld J, Abraham WT, Kar S, Lim DS, Mishell JM (2018). Transcatheter mitral-valve repair in patients with heart failure. N Engl J Med.

[4] von Bardeleben RS, Hobohm L, Kreidel F, Ostad MA, Schulz E, Konstantinides S (2019). Incidence and in-hospital safety outcomes of patients undergoing percutaneous mitral valve edge-to-edge repair using MitraClip: five-year German national patient sample including 13,575 implants. EuroIntervention.

[5] Asmarats L, Rodriguez-Gabella T, Chamandi C, Bernier M, Beaudoin J, O’Connor K (2018). Infective endocarditis following transcatheter edge-to-edge mitral valve repair: a systematic review. Catheter Cardiovasc Interv.

[6] Nicolò F, Scrofani R, Antona C (2019). Bartonella haenselae infective endocarditis following transcatheter edge-to-edge mitral valve repair: a case report. J Card Surg.

[7] Tayyar R, Khan O, Chauhan K, Ines A, Carnish E (2020). Pseudomonas MitraClip® endocarditis: a case report and review of literature. IDCases.

[8] Roslan A, Kamsani SH, Jauhari Aktifanus AT, Krishnan M, Hakim N, Megat Samsudin WN (2018). Butterfly in the heart: infective endocarditis after MitraClip procedure. CASE.

[9] Mozumder SI, Rutherford MJ, Lambert PC (2017). A flexible parametric competing-risks model using a direct likelihood approach for the cause-specific cumulative incidence function. Stata J.

[10] Hermanns H, Wiegerinck EMA, Lagrand WK, Baan J, Cocchieri R, Kaya A (2019). Two cases of endocarditis after MitraClip procedure necessitating surgical mitral valve replacement. Ann Thorac Surg.

[11] Rempfer E, Basinger H, Stawovy L, End B, Shockcor W, Minardi J (2020). MitraClip-associated endocarditis: emergency department diagnosis with point of care ultrasound. J Emerg Med.

[12] Leow K, Isreb C, Brown M (2020). MitraClip-related infective endocarditis in a frail, elderly patient: a case report. Eur Hear J Case Rep.

[13] Kadoya Y, Zen K, Fukai K, Matsubayashi K, Yamano M, Yamano T (2021). Recurrent infective endocarditis following transcatheter edge-to-edge mitral valve repair with MitraClip system. Korean Circ J.

[14] Wilson W, Taubert KA, Gewitz M, Lockhart PB, Baddour LM, Levison M (2007). Prevention of infective endocarditis: guidelines from the American Heart Association. Circulation.

[15] Habib G, Lancellotti P, Antunes MJ, Bongiorni MG, Casalta JP, Del Zotti F (2015). 2015 ESC guidelines for the management of infective endocarditis. Eur Heart J.

[16] Duval X, Alla F, Hoen B, Danielou F, Larrieu S, Delahaye F (2006). Estimated risk of endocarditis in adults with predisposing cardiac conditions undergoing dental procedures with or without antibiotic prophylaxis. Clin Infect Dis.

[17] Nishimura RA, Otto CM, Bonow RO, Carabello BA, Erwin JP, Fleisher LA (2017). 2017 AHA/ACC focused update of the 2014 AHA/ACC Guideline for the management of patients with valvular heart disease: a report of the American College of Cardiology/American Heart Association Task Force on Clinical Practice Guidelines. J Am Coll Cardiol.

[18] Otto CM, Nishimura RA, Bonow RO, Carabello BA, Erwin JP, Gentile F (2020). ACC/AHA guideline for the management of patients with valvular heart disease. J Am Coll Cardiol.

